# COMMUNITY RATES OF CAREGIVING IN RI AND CT: FINDINGS FROM THE HEALTHY AGING DATA REPORTS

**DOI:** 10.1093/geroni/igae098.2409

**Published:** 2024-12-31

**Authors:** Shan Qu, Taylor Jansen, Chae Man Lee, Nina Silverstein, Elizabeth Dugan

**Affiliations:** University of Massachusetts Boston, Boston, Massachusetts, United States; University of Massachusetts Boston, Boston, Massachusetts, United States; University of Massachusetts Boston, Boston, Massachusetts, United States; University of Massachusetts Boston, Needham, Massachusetts, United States; University of Massachusetts Boston, Boston, Massachusetts, United States

## Abstract

Informal caregivers are a significant but often overlooked part of the US healthcare system. Grandparents may provide and receive care and may be important members of the informal support network for many families. The demands and stresses of caregiving may negatively impact caregivers’ health. Understanding the prevalence of caregiving at the community level can guide resource allocation, policy decisions, and advocacy efforts to support caregivers and improve all residents’ well-being. The present study examines disparities in community rates in Rhode Island (RI) and Connecticut (CT) for three caregiving indicators: older adults aged 60+ who provided caregiving in the last month, grandparents raising grandchildren, and grandparents who live with grandchildren. For this study, rates were calculated from the following data sources: the Behavioral Risk Factor Surveillance System (2015-2017) and the American Community Survey (2016-2018). Small area estimation techniques were used to calculate age-sex adjusted community rates (https://healthyagingdatareports.org/). Results showed that while RI had a slightly higher statewide rate of caregiving (22.5% vs 21.7% in CT) the range in community rates (14.84%-27.43%) was much larger in CT compared to RI (19.17%-27.30%). RI (0.8%) also had a slightly higher rate of grandparents raising grandchildren than CT (0.7%), but Hartford CT had the highest community rate in the two states (2.93%). RI (2.8%) also had a higher rate of grandparents who live with grandchildren than CT (2.5%). This study found that older adults providing caregiving is prevalent, and older adults play a significant role in raising grandchildren in CT and RI.

